# Expression of SR-B1 receptor in breast cancer cell lines, MDAMB-468 and MCF-7: Effect on cell proliferation and apoptosis

**DOI:** 10.22038/ijbms.2021.56752.12674

**Published:** 2021-08

**Authors:** Neamat Karimi, Fatemeh Soghra Karami Tehrani

**Affiliations:** 1 Department of Clinical Biochemistry, Cancer Research Laboratory, Faculty of Medical Sciences, Tarbiat Modares University, Tehran, Iran

**Keywords:** BC, BLT-1, HDL, MCF-7, MDA-MB-468, SR-B1

## Abstract

**Objective(s):**

High-density lipoprotein (HDL) is necessary for proliferation of several cells. The growth of many kinds of cells, such as breast cancer cells (BCC) is motivated by HDL. Cellular uptake of cholesterol from HDL which increases cell growth is facilitated by scavenger receptors of the B class (SR-BI). The proliferative effect of HDL might be mediated by this receptor. It is also believed that HDL has an anti-apoptotic effect on various cell types and promotes cell growth. This study was designed to investigate SR-BI expression, proliferation and apoptotic effect of HDL on human BCC lines, MCF-7 and MDA-MB-468.

**Materials and Methods:**

Real-time-PCR method was used to evaluate expression of SR-BI, and cholesterol concentration was measured using a cholesterol assay kits (Pars AZ moon, Karaj, Iran). Cell viability was assessed using the MTT test. To identify cell apoptosis, the annexin V-FITC staining test and caspase-9 activity assay were applied.

**Results:**

Treatment of both cell lines (MCF-7, MDA-MB-468) with HDL results in augmentation of SR-BI mRNA expression and also elevation of the intracellular cholesterol (*P*<0.01). HDL induced cell proliferation, cell cycle progression, and prevented activation of caspase-9 (*P*<0.05). We also demonstrated that inhibition of SR-B1 by BLT-1 could reduce cell proliferation, and induction of SR-B1 receptor by quercetin increased HDL-induced proliferation in both cell lines (*P*<0.05).

**Conclusion:**

It can be concluded that alteration in HDL levels by SR-B1 activator (Quercetin) or inhibitor (BLT-1) may affect BCC growth and apoptosis induction.

## Introduction

Epidemiological studies have revealed a strong relationship between breast cancer (BC) and lipid disorders (1-4). Elevation of total cholesterol (5-9), VLDL-C (8, 10), LDL-C (7, 8), and reduction of HDL cholesterol (5, 8, 10-12) have been reported. However, how dyslipoproteinemia stimulates breast cancer cell (BCC) growth is not fully elucidated. HDL is a growth factor in BC that transports cholesterol to the cells via scavenger receptor class B type I, SR-BI (13-16). In addition, HDL could activate phosphoinositide-3-kinase/AKT and extracellular signaling regulated kinase (ERK) which stimulate cell growth (17-21). Up-regulation of SR-BI at protein and mRNA levels (13) has been reported in breast tumors that may mediate the pro-proliferative effect of HDL. It has been shown that the pro-proliferative effect of HDL was mediated by SR-BI. A dominant-negative mutant of SR-B1 (inactive SR-B1) can inhibit BCC proliferation. Overexpression of SR-BI has been presented in different tumor cell lines such as nasopharyngeal, ovarian, pancreatic, colorectal, breast, prostate, and liver cancers (22, 23). Expression of the HDL receptor, SR-B1, is stimulated, by quercetin (a plant flavonoid). It has been demonstrated that quercetin stimulates the PPAR_Y_/LXRa pathway (24). SR-B1 is specifically inhibited by BLT-1, block lipid transport-1 (25), which blocks HDL-CE entry by targeting cys384 in the extracellular domain of SR-B1 (26). BLT-1 suppresses the BCC proliferation mediated by SR-B1 (27). Recent studies provide evidence that HDL, through the SR-B1 receptor, can interfere in cell proliferation (28) and inhibition of cell apoptosis (29). Therefore, targeting the SR-B1 receptor and its related pathways have been introduced as a new approach in cancer treatment(27). Quercetin increases the expression of the SR-B1 receptor (24), and BLT-1 inhibits the receptor function by binding to the cysteine part of the receptor (26). Regarding the effect of HDL through SR-BI, on the cell proliferation and apoptosis of BCC lines, no comprehensive research has been reported. Therefore, in this study, SR-BI expression and the role of HDL in cell growth and apoptosis have been evaluated in estrogen receptor (+) & (-), MCF-7 and MDA-MB-468, BCC lines using SR-BI inhibitor, BLT-1 and activator, and quercetin.

## Materials and Methods

***Cell culture and chemical materials***

Streptomycin, penicillin, NaCl/PI, trypsin/EDTA, and RPMI 1640 were purchased from Gibco (Grand Island, NY, USA) and exogenous High-Density Lipoprotein (HDL) and MßCD from Sigma Aldrich. The purity of exogenous HDL was >98%. The apoptotic detection kit, Annexin-V-FITC, Propidium Iodide (PI), MTT [3-(4, 5-dimethylthiazol-2-yl)-2, 5-diphenyl tetrazolium bromide], and dimethylsulfoxide were provided by Sigma Aldrich (Munich, Germany) and MDA-MB-468 and MCF-7 cell lines were purchased from the Pasteur Institute, Iran. Cells were cultured in RPMI 1640 containing FCF (10% v/v), streptomycin (100 µg/ml), and penicillin (100 u/ml) and were kept at 37 °C with 100% humidity and 5% CO_2_. They were then trypsinized at 70-100% confluence. The effects of exogenous HDL on BCC growth were always compared with the cells treated with medium alone. A medium supplemented with 10 % fetal calf serum was used to dissolve HDL, MßCD, and BLT-1. 

***Cell viability assay***

MTT assay was used to evaluate cell viability. Briefly, cells were detached by 0.25% trypsin 0.02 % EDTA. Aliquots of 5-7.5 × 10^3 ^cells were placed in each well (200 µl) of a 96-well plate (Thermo Scientific, Germany). Cells were permitted to attach overnight and then were stimulated with different concentrations of HDL (10, 50, 100, 200, and 250 µg/ml) for 6, 12, 18, and 24 hr; quercetin (15, 30, 50, 100, and 200 µM) for 2, 4, and 6 hr; and BLT-1 (15, 30, 50, 100, and 200 nM) for 1, 3, and 5 hr. Each well was incubated with 20 µl MTT at 37 °C for 4 hr. The supernatant was then removed and in order to solubilize the blue-purple crystals of formazan, 200 µl DMSO was added to each well. The absorbance was measured at 570 nm using a microplate reader (Tecan, Austria). Each experiment was repeated 3 times, with 3 replicates. The viability was evaluated based on a comparison with untreated cells. Effect of dose values represents HDL and quercetin concentrations required to stimulate 50% of cell proliferation, and IC_50 _values represent the BLT-1 concentrations needed to inhibit 50% of cell proliferation, calculated by Graph Pad Prism 8 (Graph Pad, Inc., La Jolla, CA, USA).

The survival rate was calculated as follows: 

Survival rate = (Absorbance of treatment/ absorbance of control cell) × 100% 

***Assessment of cell death***

Annexin V/Propidium iodide staining assay was applied in order to measure cell death. Briefly, 3 × 10^3 ^cells were pretreated with 50 nM BLT-1 for 3 hr or with 25 µM quercetin for 4 hr and were then treated with 100 µg/ml HDL for 18 hr. Afterward, they were washed 2 times with PBS, mixed with 500 µl of binding buffer, stained with 5 µl of annexin V fluorescein isothiocyanate (FITC) and 5 µl of PI (50 µg/ml) in a dark place at room temperature for 10 min. Apoptotic cells were quantified using a FACS caliber flow cytometer (BD Biosciences, San Jose, CA, USA); for each sample, 1 × 10^9 ^cells were counted. Early (PI- negative, annexin V- positive) and late (double-positive of annexin V and PI) apoptotic cells were then identified. Flowing Software version 2.4.1 was used to analyze the cell percentages in each quadrant and the test was repeated at least 3 times. 

***Cell cycle analysis***

In ethanol-fixed cells, the fluorescent molecule, PI, binds to double-strand DNA in the nucleus. The PI fluorescence intensity of the cells with damaged DNA, in the sub- G1 stage, was weaker than that of the normal cells (30). In the present investigation, 3 × 10^5 ^cells were grown in 12-well plates overnight. Cells were pretreated with 50 nM BLT-1 for 3 hr or with 25 µM quercetin for 4 hr and then they were treated with 100 µg/ml HDL for 18 hr. Cells were washed with PBS, twice, and resuspended in 0.3 ml PBS. For fixation of the cells, 0.7 ml of cold ethanol (70 %) was added for at least 3 hr on ice. Cells were washed with PBS and resuspended in 0.25 ml of PBS containing 5 µl of RNase A (10 mg/ml) and Triton ×-100 (0.1 %). They were incubated at 37 °C for 1 hr and 10 µl of PI (50 µg/ml) was added. An FACS caliber flowcytometer was used to determine the DNA content of the cell. The percentage of apoptotic cells comprising the sub-G1 population and non-apoptotic cells was determined at each phase of the cell cycle. Flowjo ver. 10. 0. 8 (FlowJo LLC) was used for analysis of cytographs. 

***Caspase-9 activity assay***

ELISA kit (eBioscience) was used for detection of Caspase-9 activity. Briefly, the cells were seeded in 75-cm^2^ flasks at a density of 5 × 10^6 ^cells/flask. In a 96-well plate, cultured cells were pretreated with 50 nM BLT-1 or 25 µM quercetin, prior to treatment with 100 µg/ml HDL. The cells were trypsinized, collected, washed with PBS, and underwent lysis in the presence of lysing buffer, at room temperature for 60 min and centrifuged at 1000 ×g for 15 min. The next steps have been done according to the manufacturers’ instructions, and absorbance was measured at 450 nm using a plate reader. 

***Real-time PCR***

Using TRIzol reagent, total RNA was extracted. With AffinityScript and oligo (DT) primers to cDNA synthesis (Parstous), RNA was reverse transcribed (Parstous Kit). A real-time PCR system (Bio-Rad) was used to analyze synthetic genes quantitatively. Real-time PCR was performed in a 20-µl reaction volume with 1 µM of each primer and 10 µl of SYBR Green Real-time PCR master mix (Parstous Kit). Quantitative Real-Time PCR tests were performed in duplicate for each sample. Relative mRNA abundance was normalized to GAPDH (forward primer 5^’^-GAAGGTGAAGGTCGGAGTC-3^’ ^and reverse primer 5^’^-GAAGATGGTGATGGGATTTC-3^’^). The following primers were used for SR-B1 (reverse primer 5^’^-ACTGAACCTGCAGGTGCTGA-3^’^ and forward 5^’^-ATGATCGTGATGGTGCCGTC-3’). Negative controls contained no transcript or reverse transcriptase. After amplification, melting-curve analysis was performed to verify the viability of amplicons. Quantitative measurements were carried out using the ^∆∆ ^C_t _method, and expression of GAPDH was used as the internal control. 

***Cell treatment***

To achieve 60-70% confluency, MDA-MB-468 and MCF-7 cells were seeded in 10 cm^2^ plates (2 × 10^6 ^cells/well) and incubated at 37 °C; then washed with PBS, pre-treated with BLT-1 (50 nM for 3 hr) or quercetin (25 µM for 4 hr) and then treated with HDL (100 µg/ml) for 18 hr. After washing with PBS, they were harvested by trypsin and stored at – 80 °C. 

***Isolation of cytosolic fraction***

The treated cells were re-suspended in 50 mM HEPES (pH 7.4), 10 mM NaCl, 5 mM MgCl_2_, and 0.1 mM EDTA supplemented with protease inhibitor cocktail. They were lysed by three strokes of sonication (10 sec each) with a sonicator (Misonix 3000, Farmingdale, NY, USA). The lysates were centrifuged at 14000 ×g for 20 min (4 °C), the supernatant (cytosol) was collected and stored at -80 °C for further investigation (31). 

***Cholesterol assessment***

The cholesterol content of cytosol was measured using cholesterol assay kits (Pars AZ moon, Karaj, Iran). Cholesterol level was assessed based on a standard graph (Merck, Darmstadt, Germany).

***Statistical analysis***

Graph pad Prism 8 (CA, USA) was applied to calculate the IC_50 _value. A one-way ANOVA test was applied to compare the results followed by Dunnett^`^s *post hoc* test. In all cases, mean ± SD of at least 3 independent experiments were presented, and *P*<0.05 was considered as the level of significance. 

## Results

***The effect of HDL on cell growth***

**Figure 1:**
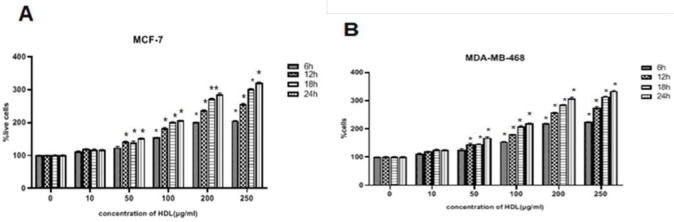
Exogenous high-density lipoprotein (HDL) induced cell growth in breast cancer cell (BCC) lines

MDA-MB-468 and MCF-7 cells were treated with HDL (10, 50, 100, 200, and 250 µg/ml) for 6, 12, 18, and 24 hr. The viability of MCF-7 and MDA-MB-468 cells was stimulated significantly by HDL (Figures 1A, B) in a dose and time-dependent fashion (*P*<0.05). Treatment with HDL, 105 µg/ml for MCF-7 and 95 µg/ml for MDA-MB-468 cells resulted in a two-fold enhancement in cell viability after 18 hr, in a dose and time-dependent fashion. The concentration of 100 µg/ml has been used in the subsequent experiments. 

***The effect of quercetin on the cell growth***

**Figure 2:**
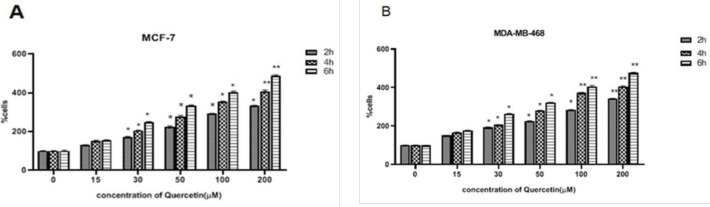
Quercetin-induced cell growth in breast cancer cell (BCC) lines

MCF-7 and MDA-MB-468 cells were treated with various concentrations of quercetin. Quercetin significantly stimulated the viability of both MCF-7 (Figure 2A) and MDA-MB-468 (Figure 2B) cells in a dose and time-dependent fashion (*P*<0.05). Treatment of MCF-7 and MDA-MB-468 cells with 30 µM and 20 µM of quercetin showed a two-fold enhancement in the rate of cell viability after 4 hr, in a dose and time-dependent fashion. In the subsequent experiments, 25 µM was applied as the optimum level. 

***The anti-proliferative effect of SR-B1 inhibitor, BLT-1***

**Figure 3:**
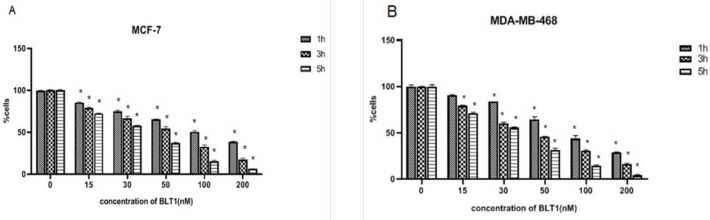
Blocks lipid transport (BLT)-1 induced cell death in breast cancer cell (BCC) lines. MCF-7 (A) and MDA-MB-468 (B) cells were treated with various concentrations of BLT-1 (15-200 µM) for 1, 3, and 5 hr. The viability of cells was detected by MTT

Effects of BLT-1 on cell viability were examined. MDA-MB-468 and MCF-7 cells were treated with various concentrations of BLT-1 (15, 30, 50, 100, and 200 nM) for 1, 3, and 5 hr. The viability of both MCF-7 (Figure 3A) and MDA-MB-468 was significantly inhibited by BLT-1 (Figure 3B) in a dose- and time-dependent fashion (*P*<0.05). After 3 hr treatment, IC50 values (the effective dose of BLT-1 inhibits 50% of growth) for MCF-7 and MDA-MB-468 cells were 55 nM and 48 nM, respectively. In the subsequent experiments, 50 nM was used as the optimum concentration. 

***Inhibition of cell apoptosis by HDL***

**Figure 4:**
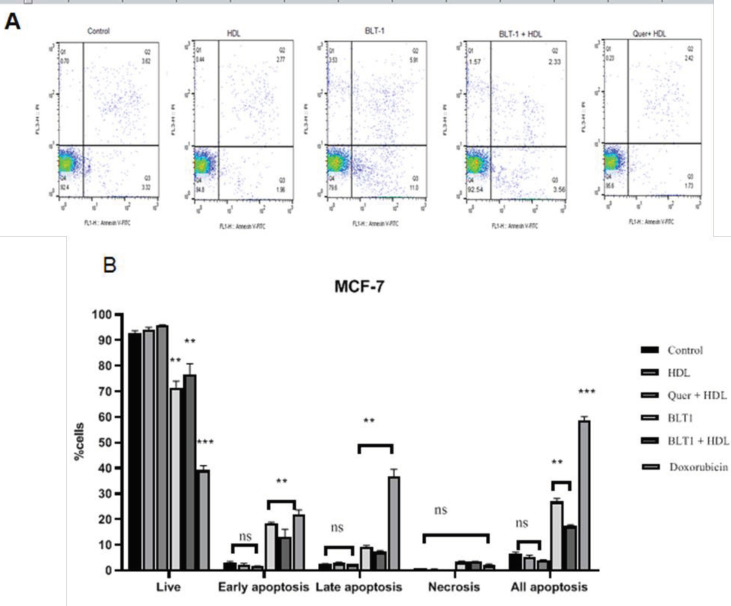
Exogenous high-density lipoprotein (HDL) inhibited apoptosis in the MCF-7 breast cancer cell (BCC) line

**Figure 5:**
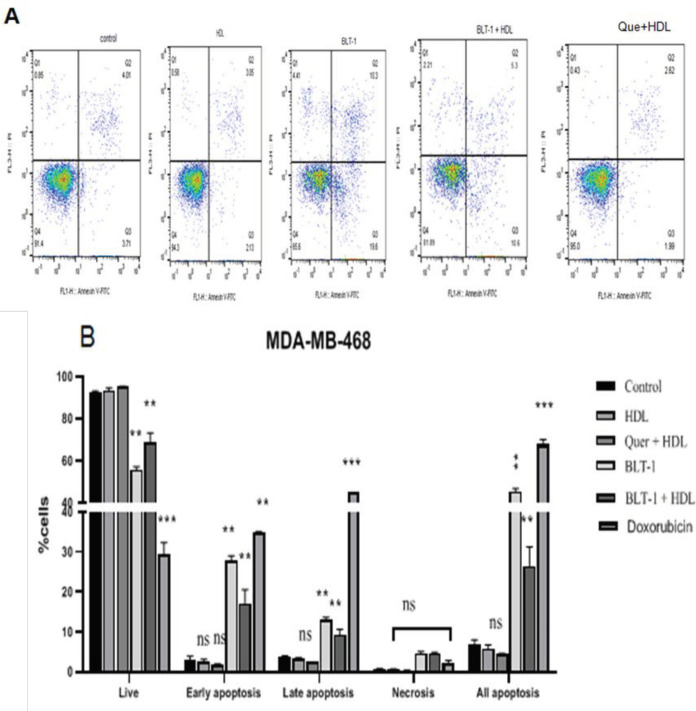
Exogenous high-density lipoprotein (HDL) inhibited apoptosis in the MDA-MB-468 breast cancer cell (BCC) line

Cells were incubated with 100 µg/ml HDL for 18 hr and the rate of apoptosis was measured. Control cells were negative for both PI and annexin V-FITC (Figure 4A). No significant difference was observed in the percentage of apoptosis in the BCC lines (*P*>0.05) after treatment with HDL (100 µg/ml) (Figure 4B). Apoptosis was optimally induced when BLT-1 was used (*P*<0.01) (Figures 4A, 5A). To describe the cell death modes by HDL, MCF-7 and MDA-MB-468 cells were pretreated with BLT-1 (50 nM) and then treated with HDL (100 µg/ml) for 18 hr. Taken together, HDL inhibited apoptosis that was observed in the presence of BLT-1 (*P*<0.05) (Figures 4A, 5A). The early apoptotic stage of HDL co-incubated with BLT-1 decreased significantly (*P*<0.05) (Figure 4B). This suggests that HDL effectively blocked BLT-1 induced apoptosis. However, no significant difference was seen between the rate of apoptosis in quercetin/HDL treated (MCF-7 and MDA-MB-468) and control cells (*P*>0.05) (Figures 4B, 5B). 

***Inhibition of BLT-1 by HDL on the cell***

**Figure 6:**
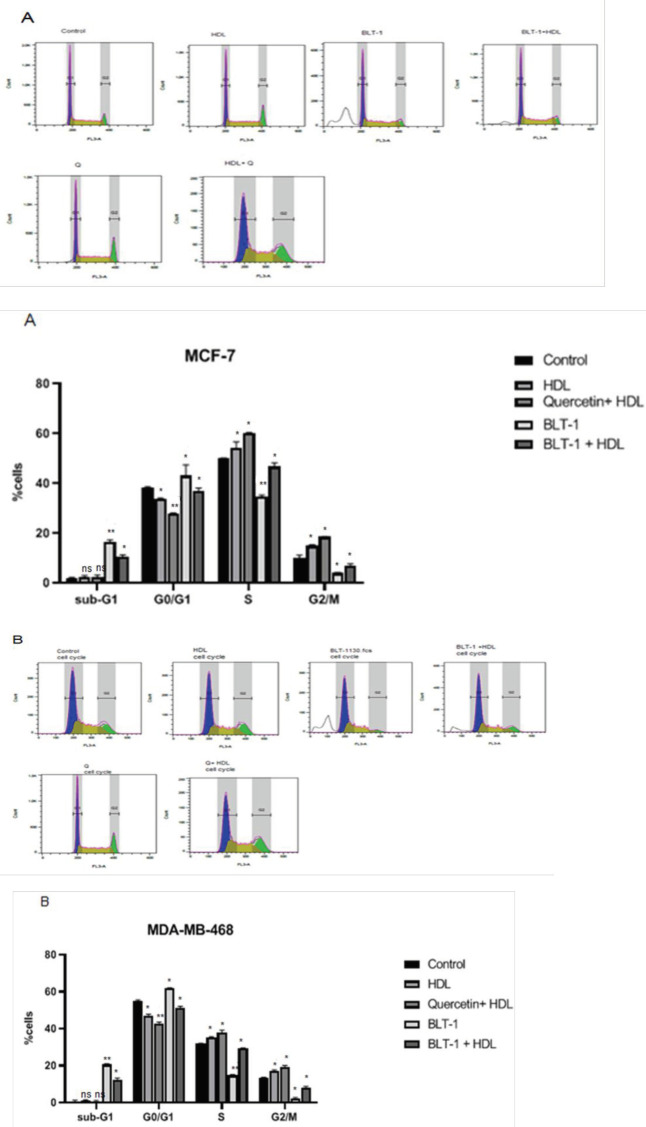
Exogenous high-density lipoprotein (HDL) induces cell cycle to stimulate and inhibit apoptosis in MCF-7 (A) and MDA-MB-468 (B) cell lines. DNA content of treated cells with exogenous HDL for 18 hr. B. Analysis of cell cycle distribution in cells after treatment with HDL

Exposure of MCF-7 to BLT-1 (50 nM) in comparison with untreated cells for 3 hr induced an alteration in the cell cycle distribution, especially BLT-1 showed an enhancement in the sub-G1 and a reduction in the G2/M phases (*P*<0.05) (Figure 6A). The co-incubation with HDL completely blunted the BLT-1-induced shift from G2/M to sub-G1 phase (*P*<0.01) (Figure 6A). After exposure to BLT-1, MDA-MB-468 cells also displayed an enhancement in the cell percentages in the sub-G1 phase (*P*<0.05) (Figure 6B). Co-incubation with HDL restored the basal cell cycle profile in comparison with the BLT-1-treated cells; i.e., those in the G2/M phase were increased and those in the sub-G1 phase were decreased (*P*<0.01) (Figure 6B). HDL alone affected the cell cycle profile of MCF-7 and MDA-MB-468 cells. It resulted in an increase in S and G2/M phases and a decrease in G0/G1 phase. This effect was outstanding while the cells were co-incubated with quercetin (*P*<0.05) (Figures 6A, 6B). 

***Suppression of caspase-9 activity by exogenous HDL***

**Figure 7:**
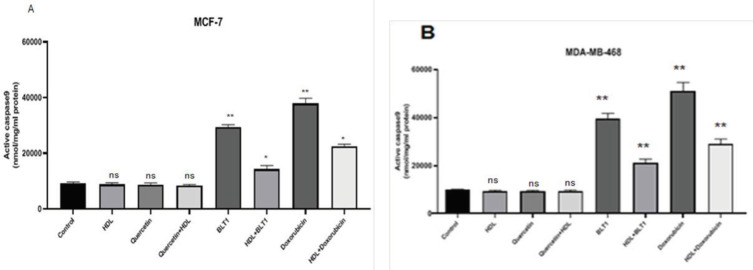
Specific activity of caspase-9 in MCF-7 (A) and MDA-MB-468 (B) cell lines. Caspase-9 specific activity was increased with Blocks lipid transport (BLT)-1 (** *P*<0.01) and decreased by BLT-1- HDL (* *P*<0.05)

Following treatment with 100 µg/ml of HDL, there were not any significant differences in the activity of caspase-9 in either cell line, (*P*>0.05)., Caspase-9 stimulation by BLT-1 (50 nM) or doxorubicin (10 µM) was suppressed by HDL in both cell lines (*P*<0.05) (Figures 7 A, B). 

***Expression of SR-BI***

**Figure 8:**
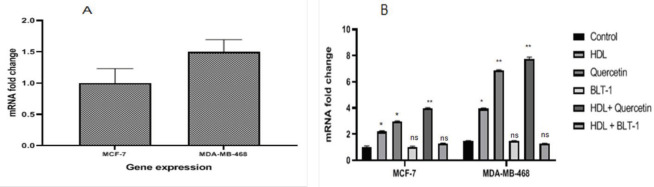
(A) SR-BI mRNA detection in human BCC lines, MCF-7 and MDA-MB-468. (B) Real-time PCR analysis of SR-BI mRNA expression in MCF7 and MDA-MB-468 cells following BLT-1(50 nM) or quercetin (25 µM) pretreatment in the presence or lack of HDL (100 µg/ml) for 18 hr. The expression rates were normalized to human GAPDH mRNA

Effects of HDL or SR-BI activator and quercetin on mRNA expression of SR-BI were studied. The results indicated that SR-BI expression level was increased (*P*<0.05) (Figure 8A). The effect of SR-BI inhibitor, BLT-1, on the mRNA expression of SR-BI was examined and the result indicated that SR-BI expression level did not alter in either cell line compared with that of the untreated group (*P*>0.05) (Figure 8B). 

***Regulation of intracellular cholesterol*** ***by SR-B1 receptor***

**Figure 9:**
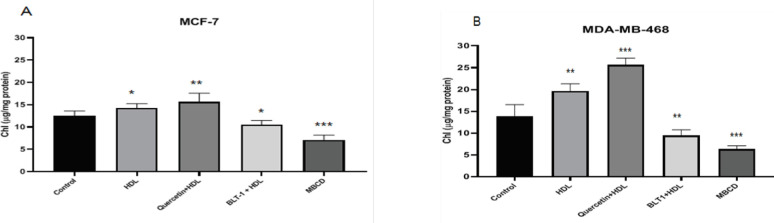
High-density lipoprotein (HDL)effects on total cholesterol levels in breast cancer cell (BCC). BCC lines MCF-7 (A) and MDA-MB-468 (B) pretreated with BLT-1 (50 nM) or Quercetin (25 µM) and MBCD (1.25 µM) after treatment with HDL(100 µg/ml) for 18 hr. Total cholesterol was measured

To assess the role of the SR-B1 receptor in the regulation of cholesterol homeostasis, we evaluated the effect of SR-B1 inhibition or overexpression on the cellular cholesterol levels. After inhibition of SR-B1 receptor, a significant decrease and after overexpression of SR-B1 receptor, a significant increase was observed in the cellular content of cholesterol in comparison with control cells (*P*<0.05). Unlike Methyl-ß-cyclodextrin (MBCD), which lowered total cellular cholesterol levels (*P*<0.001), HDL increased total cholesterol in both cell lines, notably in MDA-MB-468 cells (*P*<0.01) (Figures 9A and 9B). 

MCF-7 (A) and MDA-MB-468 (B) cells were treated with various concentrations of exogenous HDL (10-250 µg/ml) for 6, 12, 18, and 24 hr. MTT

assay was applied to determine cell viability. Cell-growth induction occurred in a dose and time-dependent fashion in both cell lines The data

represents the Mean ± SD of three separate experiments. Statistical differences were analyzed by ANOVA (*P<0.05)

MCF-7 (A) and MDA-MB-468 (B) cells were treated with various concentrations of quercetin (15, 30, 50, 100, and 200 µM) for 2, 4, and 6 hr. MTT

assay was applied to determine cell viability. Cell-growth induction occurred in a dose and time-dependent fashion in both cell lines. The data

represents the Mean ± SD of three separate experiments. Statistical differences were analyzed by ANOVA; * *P*<0.05; ** *P*<0.01

A. Exogenous HDL (100 µg/ml) treated MCF-7 with or without quercetin (25 µM) or Blocks lipid transport (BLT)-1 (50 nM) for 18 hr. Cells were

pretreated with Quercetin (4 hr) and BLT-1 (3 hr) before treatment with HDL. B. Incubation with HDL or quercetin, (18 hr), did not affect early

or late apoptosis. The apoptosis was significantly induced by treatment with BLT-1. Induction of apoptosis by BLT-1 was blocked by treatment

with HDL in BCC. Data are representative of three independent experiments. * *P*<0.05, ** *P*<0.01, and ***P<0.001 compared with control. NS: not

significantly different from untreated cells

A. Exogenous HDL (100 µg/ml) treated MCF-7 with or without quercetin (25 µM) or BLT-1 (50 nM) for 18 hr. Cells were pretreated with Quercetin (4 hr) and Blocks lipid transport (BLT)-1 (3 hr) before treatment with HDL. B. Incubation with HDL or quercetin (for 18 hr) did not affect early or late apoptosis. Apoptosis was significantly induced by treatment with BLT-1. Induction of apoptosis by BLT-1 was blocked by treatment with HDL in BCC

## Discussion

A transmembrane protein named SR-B1 could specifically bind to HDL to simplify the cellular transport of cholesterol (32). Various tumor cell lines express SR-B1 (23, 33-35). In the current study, a high expression of SR-B1 was seen in cell lines. We found that the expression of SR-B1 was increased by treatment with HDL or quercetin (Figure 8B). After administration of HDL or quercetin, a significant diversity was observed in SR-B1 mRNA expression of MCF-7 and MDA-MB-468 cells (Figure 8B), although we found no significant differences after inhibition with BLT-1. In the mammalian cells, HDL and SR-B1 receptors mediate important functions such as inhibition of apoptosis (36), induction of endothelial cell migration (37), and proliferation of prostate cancer cells (38). Malignant cells can utilize the SR-B1/HDL pathway to take up cholesteryl ester. It has been shown that in prostate and BCs, the expression of SR-B1 is up-regulated. (22, 39). Among these, the roles of HDL/SR-B1 have been most studied. The HBL-100 cell line can take up cholesteryl ester from apoE- depleted HDL_3 _via SR-B1 (40). In addition, overexpression of SR-B1 stimulates HDL-mediated proliferation of MCF-7 cells through the PI_3_k/AKT pathway (13). Conversely, overexpression of mutant SR-B1 (lacking the C-terminal residues 465-509) has been displayed to hamper proliferation induced by HDL in the MCF-7 cell line (13). The mutation in SR-B1 suppressed SR-B1 expression and also inhibited the induction of BC in mice (13). The precise role of SR-B1/HDL in the regulation of BCC growth encouraged us to investigate the impact of SR-B1/HDL on cell growth and apoptosis in BC. Treatment of MCF-7 and MDA-MB-468 cells with increasing concentration of HDL provoked cell growth in a time- and dose-dependent manner in both cell lines. Our data is consistent with the stimulatory effect of HDL on the growth of the prostate (41) and BCC lines (42). The expanded reaction of MCF-7 and MDA-MB-468 cells to HDL-induced cell proliferation seemed to relate to their more significant level of SR-B1, proposing a part of SR-B1 in these processes. It was confirmed by BLT-1(inhibitor of SR-B1) experiments and it tests that decreased SR-B1 expression. The results showed that the impact of HDL on the BCC growth is related to an increase in G2/M percentage and decrease in G1/G0 cell population, which is consistent with those shown in cell cycle progression (43) and vascular smooth muscles cells (44). Our findings exhibited that the growth stimulation mediated by quercetin/HDL was accompanied by a remarkable increase in the growth of two BCC lines. In this study, the impact of quercetin/HDL on BCC growth was related to an increase in the percentage of G2/M cell population, which is consistent with the efficient role of quercetin in up-regulation of SR-B1 receptor shown in the liver cancer cell lines (24). Cell cycle analysis showed that when the cells were treated with HDL alone, for 18 hr, the cell population in S and G2/M phases was increased. There was no difference in the sub G1 cell population. However, when BCC were treated with BLT-1, the percentage of Sub-G1 cells was increased, indicating cell apoptosis. To confirm the anti-apoptotic effect of HDL, the cells were pretreated with BLT-1 for 3 hr and then treated with HDL for 18 hr. The population of cells in the sub-G1 phase was reduced significantly when compared with those treated with BLT-1 alone. Pretreatment of MCF-7 and MDA-MB-468 cells with quercetin for 4 hr followed by incubation with HDL for 18 hr, resulted in cell transition from the G1 phase to S and G2/M phases, which was more effective than HDL alone. In fact, elevation of HDL stimulates further entry of the cells from G1 to S and G2/M phases, and reduction of HDL increased apoptotic cells, the cells in sub-G1 phase. These results confirm that the SR-B1 receptor mediates the effects of HDL on the cell cycle distribution in BC. We demonstrated that HDL potently protects against apoptosis and examined the effect of BLT-1 (Block lipid transport-1), an inhibitor of SR-B1, on the BCC lines. In this study, we exhibited that the effect of BLT-1 on BCC growth is related to an enhancement in the rate of the sub-G1 population which is in line with the effective role of SR-B1 in BCCs(27). Cell growth inhibition by BLT-1 was accompanied by significant induction of apoptosis in BCC lines. However, pretreating with BLT-1 and treating with HDL, the percentage of apoptotic cells decreased significantly. In addition, flowcytometry results showed that treatment with exogenous HDL did not have a dramatic impact on the rate of apoptotic cells. To prove the anti-apoptotic effect of HDL, we also measured caspase-9 activity in the treated cells with HDL and found no significant differences. However, treatment of the cell with BLT-1 alone increased caspase-9 activity which indicates that BLT-1 induces apoptosis. When cancer cells were pretreated with BLT-1 for 3 hr and then treated with HDL, caspase-9 activity decreased significantly. This finding confirms that exogenous HDL inhibits apoptosis in both cell lines and the inhibition is induced by reduction in the activation of the mitochondrial apoptotic pathway. The present results are in line with those of Michalides R, 1999 of investigation on the effect of HDL on BCCs (43). The interaction between HDL and receptor, SR-B1 is a key stage in HDL-mediated regulation of cell cholesterol content, but document offers its relationship in many other atheroprotective activities of HDL (44, 45). In addition, the role of cholesterol in tumor progression has extensively been evaluated in different cancers. (46). Cholesterol regulates essential signaling pathways in cell proliferation, migration, survival and thereby promoting cancer progression(47-49). Furthermore, cellular cholesterol homeostasis is also regulated by the SR-B1 receptor (50). Our present data demonstrated that quercetin and BLT-1 could alter the cholesterol content of two examined BCC lines. This investigation recommends a critical role for cholesterol in the regulation of cell proliferation. Some evidence showed that elevation in the cellular cholesterol level induces cell proliferation (51) and, on the contrary, reduction in the cholesterol level has the opposite effect (52, 53). Our findings also demonstrated that inhibition of SR-B1 in BCC lines is related to reduction of cellular cholesterol level and cell proliferation, which is consistent with the results of Danilo *et al*., 2013 in which down-regulation of SR-B1 in BCC had a relationship with the reduction of cholesterol content (27). To characterize whether SR-B1/HDL mediated growth stimulation depends on the estrogen; MCF-7 and MDA-MB-468 BCC lines were applied in the study as estrogen receptor (+) & (-), respectively. A significant difference was observed in the sensitivity of the cells to the effect of SR-B1/HDL. Reports on the effect of SR-B1/HDL on cancer cell growth are controversial. An *in vitro* study showed that the growth of tumor cells was directly related to an increase in HDL (54). It has been reported that HDL increased cell proliferation in ER^- ^BCC (42), although, another study found no effect on the proliferation of ER^-^, PR^-^, HER^-^ (MDA-MB -231) BCC (55) and another showed a reduction in the number of viable cells (56). In addition, it has been demonstrated that HDL increased the proliferation of ER^+ ^when compared with that of ER(-) BCC (57). An inverse relation between HDL levels and cell proliferation and metastasis has been reported (58), although, the tumorigenic effect of HDL in cancer cells has been supported (59, 60) This conflicting data on the important role of SR-B1/HDL in the regulation of cell growth could be clarified by tissue specificity or other possible molecular mechanisms that are probably involved in the SR-B1 signaling pathway. These results recommend that future investigations on the impact of HDL may reveal new routes for prevention and treatment of BC.

## Conclusion

It can be concluded that HDL and SR-B1 can stimulate cell proliferation and also inhibit cell apoptosis in two BCC lines. Inhibition of SR-B1 could prohibit cell growth and induce apoptosis. These findings may suggest HDL and SR-B1 as therapeutic agents in BC treatment.
